# Interleukin-10 deficiency exacerbates inflammation-induced tau pathology

**DOI:** 10.1186/s12974-021-02211-1

**Published:** 2021-07-18

**Authors:** Lea L. Weston, Shanya Jiang, Devon Chisholm, Lauren L. Jantzie, Kiran Bhaskar

**Affiliations:** 1 Department of Molecular Genetics and Microbiology, University of New Mexico, MSC08 4660, 1 University of New Mexico, Albuquerque, NM 87131 USA; 2 Department of Pediatrics, Johns Hopkins University School of Medicine, Baltimore, MD 21287 USA; 3 Department of Neurology, University of New Mexico, 1 University of New Mexico, Albuquerque, NM 87131 USA

**Keywords:** Alzheimer’s disease, Tau, Microglia, Neuroinflammation, Interleukin-10

## Abstract

**Background:**

The presence of hyperphosphorylated microtubule-associated protein tau is strongly correlated with cognitive decline and neuroinflammation in Alzheimer’s disease and related tauopathies. However, the role of inflammation and anti-inflammatory interventions in tauopathies is unclear. Our goal was to determine if removing anti-inflammatory interleukin-10 (IL-10) during an acute inflammatory challenge has any effect on neuronal tau pathology.

**Methods:**

We induce systemic inflammation in *Il10-*deficient (*Il10*^*−*/*−*^) versus *Il10*^*+*/+^ (Non-Tg) control mice using a single intraperitoneal (i.p.) injection of lipopolysaccharide (LPS) to examine microglial activation and abnormal hyperphosphorylation of endogenous mouse tau protein. Tau phosphorylation was quantified by Western blotting and immunohistochemistry. Microglial morphology was quantified by skeleton analysis. Cytokine expression was determined by multiplex electro chemiluminescent immunoassay (MECI) from Meso Scale Discovery (MSD).

**Results:**

Our findings show that genetic deletion of *Il10* promotes enhanced neuroinflammation and tau phosphorylation. First, LPS-induced tau hyperphosphorylation was significantly increased in *Il10*^*−*/*−*^ mice compared to controls. Second, LPS-treated *Il10*^*−*/*−*^ mice showed signs of neurodegeneration. Third, LPS-treated *Il10*^*−*/*−*^ mice showed robust IL-6 upregulation and direct treatment of primary neurons with IL-6 resulted in tau hyperphosphorylation on Ser396/Ser404 site.

**Conclusions:**

These data support that loss of IL-10 activates microglia, enhances IL-6, and leads to hyperphosphorylation of tau on AD-relevant epitopes in response to acute systemic inflammation.

## Background

Alzheimer’s disease (AD) is one of the tauopathies and the most common neurodegenerative disease involving the abnormal phosphorylation and accumulation of the microtubule-associated protein tau (MAPT or tau). Tau is a neuron-enriched protein known to bind and stabilize microtubules under homeostatic conditions and has non-microtubule-binding functions [1, 2]. However, in tauopathies, tau acquires abnormal toxic forms via various post-translational modifications that lead to loss of tau’s function or gain of toxic function [2–4]. The presence of hyperphosphorylated tau has been shown to strongly correlate with cognitive decline [5–7] and neuroinflammation [8, 9]. Increasing evidence suggests neuroinflammation is a major component of tauopathies including AD [10, 11]. Additionally, previous studies suggest that activated (pro-inflammatory) microglia are sufficient to induce hyperphosphorylation of tau both in vivo and in vitro [12]. Furthermore, in vivo animal models suggest that inflammation precedes and contributes to tau pathology [12]. Therefore, understanding the role of anti-inflammatory molecules is important in thwarting excessive neuroinflammation that contributes to tau pathology relevant to AD and related tauopathies.

Interleukin-10 (IL-10) is a well-establishedanti-inflammatory cytokine with an important role in preventing unrestrained inflammatory responses from activated immune cells [13, 14]. The central nervous system (CNS) resident cells, microglia and astrocytes, are both major producers of IL-10, in vitro [15]. However, a recent single cell transcriptome analysis of IL-10 production in microglia raises questions about whether microglia produce IL-10 after endotoxin challenge, in vivo [16]. IL-10 plays a role in downregulating inflammatory responses in a variety of cell types [17] including microglia in the brain [18]. Furthermore, a known single nucleotide polymorphism in the promoter region of *Il10* is associated with abnormal hippocampus-dependent memory function [19] and increased risk for AD in human subjects [20–22]. Therefore, the potential of IL-10 in reducing brain inflammation and subsequent tau pathology is understudied and important to determine in the context of AD. Interestingly, a compelling study by Chackrabarty et al. has suggested that adeno-associated viral vector-mediated overexpression of IL-10 in amyloid-precursor protein (APP) mouse model of AD exacerbated amyloid pathology and cognitive impairment due to stunted phagocytic ability of microglia [23, 24]. However, it is still unclear whether or not IL-10 plays any regulatory role in tau pathology. Based on this, we hypothesized that IL-10 plays a role in regulating the pro-inflammatorypathway that contributes to tau phosphorylation within the brain.

In the current study, we sought to determine if IL-10 suppresses inflammation leading to tau pathology in a model of acute systemic inflammation induced by lipopolysaccharide (LPS). We have shown that LPS-induced microglial activation promotes hyperphosphorylation of tau in non-transgenic mice through toll-like receptor 4 (TLR4) signaling. In our previous study, 1 mg/kg b.w. of LPS did not induce significant increases in AT8 or AT180 tau phosphorylation compared to vehicle-treated controls, and even 10 mg/kg b.w. of LPS had only a moderate increase in pTau in the wild-type mice [25]. This suggests a homeostatic control mechanism potentially regulated by an anti-inflammatory cytokine, such as IL-10, may exist. However, mice lacking microglial fractalkine receptor (CX3CR1) showed robust microglial activation and increased tau phosphorylation after systemic LPS treatment, supporting the importance of microglial involvement in driving tau pathology. Notably, the microglia-induced neuroinflammation and tau pathology was mediated via interleukin-1β, (IL-1β) and activation of p38 mitogen-activated protein kinase (p38 MAPK) pathway [12]. Here, to determine the contributions from IL-10 in regulated microglia-mediated tau pathology, we compared inflammatory responses in the brains of *Il10-*deficient (*Il10*^*−*/*−*^) versus *Il10*^*+*/+^ (Non-Tg) control mice using a moderate dose of LPS to induce systemic inflammation. We examined microglial activation and phosphorylation of tau in the hippocampi after 24 h of LPS treatment. We predicted that lack of IL-10 would exacerbate an ongoing inflammatory response to promote hyperphosphorylation of tau.

## Methods

### Animals

B6.129P2-*Il10*^*tm1Cgn*^/J (*Il10*^*−*/*−*^) [26] and C57BL/6J mice (Non-Tg) were both in the C57BL/6J genetic background obtained from the Jackson Laboratory (JAX stock #002251 and #000664). All experimental protocols involving animals were performed in accordance with the US National Institutes of Health guidelines on animal care and were approved (16-200428-BHSC; 15-200352-HSC) by the Institutional Animal Care and Use Committees of the University of New Mexico.

### Lipopolysaccharide administration

Vehicle (Veh, Hank’s balanced saline solution, HBSS) or lipopolysaccharide (LPS, Sigma #L2880) from *Escherichia coli* 055:B5 was administered (3 mg/kg body weight, b.w.; intraperitoneally, i.p.; single dose) to 5–6-month-old non-transgenic or *Il10*^*−*/*−*^ mice on C57BL/6J background. After 24 h, the mice were sacrificed, and brain tissue was collected as described below. LPS concentration is based on previous papers showing the efficacy of this dose to induce tau phosphorylation in the hippocampi of wild-type mice [27].

### Brain tissue collection and preparation

Mice were anesthetized, transcardially perfused with 0.125 M phosphate buffer (PB) and brains were removed. Left hemispheres were fixed in 4% paraformaldehyde in PB (4% PFA/PB), incubated in cryoprotection solution for 24 h, sliced into 30μm thick sagittal brain sections, and stored at -20°C in cryostorage solution until use in TUNEL and immunohistochemical analysis. Right hemispheres were micro-dissected into cortex, hippocampus, and the rest of the brain, wet weights were recorded, and the tissues were snap frozen in liquid nitrogen. The cortices and hippocampi were used in biochemical protein analysis.

### Multiplex electrochemiluminescent immunoassay

Cortical tissue sections were homogenized in a buffered sucrose solution containing protease and phosphatase inhibitors and processed for protein analysis as previously described [28–30]. Tissue homogenates were spun at 4200×*g* at 4 °C for 10 min. Protein concentrations of cortical lysates were determined by Bradford protein assay (Biorad). A total of 200 μg of protein/sample in duplicate was used to determine cytokine levels using multiplex electrochemiluminescent immunoassay (MECI). The V-PLEX Plus Proinflammatory Panel 1 (mouse) electrochemiluminescent immunoassay platform (MesoScale Discovery #K15048G, MesoScale Diagnostics LLC) was used to detect multiple cytokines, including IL-10, IL-12p70, IL-1β, TNF-α, IL-6, and the chemokine, CXCL1. Plates were read on a Quickplex SQ 120 Imager.

### Western immunoblotting

Hippocampal tissues were homogenized in Tissue-Protein Extraction Reagent, TPER (ThermoScientific, #78510) with protease and phosphatase inhibitor cocktails. The tissue homogenates were sonicated and spun at 13,700×*g* at 4 °C for 30 min. The soluble fraction was diluted with NuPage LDS Sample Buffer and Sample Reducing Agent, run on 4–12% Bis-Tris Novex NuPage gels (Invitrogen) for 1 h. Proteins were transferred from the gel to PVDF membranes overnight. Membranes were washed and blocked in 5% milk/PBS blocking buffer and incubated overnight in primary antibodies. Membranes were washed and incubated for 1 h in corresponding HRP-conjugated secondary antibodies (Jackson ImmunoResearch Laboratories, Inc. #115-035-146 and #111-035-144). Membranes were incubated with ECL reagent (ThermoScientific) and exposed to X-ray film. Immunoreactive bands on the developed film were scanned and quantified with AlphaEaseFC^TM^ Software (Alpha Innotech Corporation).

For in vivo experiments: Western blots were probed for phosphorylated tau antibodies: PHF-1 (1:10,000, a generous gift from Dr. Peter Davies); AT180 (1:5000), AT8 (1:10,000), and total tau Tau5 (1:10,000) (Thermo Scientific); and activated phospho-p38 MAPK (1:500) and total p38 MAPK (1:1000) (Cell Signaling); and loading control, glyceraldehyde-3-phosphate dehydrogenase (GAPDH) (1:20,000; Millipore).

For in vitro experiments: Western blots were probed for phosphorylated tau antibodies: PHF-1 (1:5,000); AT8 (1:5,000); Tau5 (1:5,000); GAPDH (1:10,000), activated phospho-p38 MAPK (1:500) and total p38 MAPK (1:1000) (Cell Signaling); and activated phospho-STAT3 and STAT3 (1:2000, Cell Signaling) to verify intracellular neuronal signaling downstream of IL-6.

### Immunohistochemistry

Free-floating sections were processed via standard immunohistochemical methods. Briefly, a sodium citrate solution was used for antigen retrieval, endogenous peroxidases were blocked with 3% H_2_O_2_ in PBST, and 5% normal goat serum in Triton/PBS was used as blocking buffer. Primary antibody incubation was overnight at 4°C. Incubation with corresponding Biotinylated IgG secondary antibodies (Jackson ImmunoResearch Laboratories, Inc. #115-065-003 and #111-065-144) was for 1 h. Sections were then incubated with Avidin:Biotinylated enzyme complex reagent and developed using SIGMA*FAST*^*TM*^3,3′-Diaminobenzidine (DAB) tablets (Sigma, D4293) without metal enhancer to reveal immunoreactive signals. IHC brain sections were probed for phosphorylated tau, AT180 (1:250, Thermo Scientific), and microglia, ionized calcium-binding adapter molecule 1 (Iba1) (1:500, Wako).

### TUNEL assay

For terminal deoxynucleotidyl transferase dUTP nick end labeling (TUNEL) assay, staining was performed using the protocol supplied with a Roche in situ cell death detection kit (Roche Applied Science # 11684809910). Sections were co-stained with NeuN (1:250) (Cell Signaling) to visualize neurons. The percent area of TUNEL+ staining of pyknotic neuronal bodies was imaged and quantified using fluorescent microscopy and threshold analysis in ImageJ.

### Quantifying microglia morphology

ImageJ Skeleton Analysis was based on procedures previously described [31]. Briefly, fixed brain sections were prepared by immunohistochemistry and stained with Iba1, as described above. Brightfield photograph images (40×) of Iba1-stained sagittal brain sections were processed into binary skeletonized images using ImageJ with the following steps. First, images were run through a bandpass filter and converted to grayscale, automatic brightness/contrast was applied, followed by the Unsharp Mask filter and Despeckle tools for removing noise. Auto-threshold was applied to create a black and white image contrast of the microglia. An additional despeckle step followed by “binary-close” and “noise-remove outliers” functions (as described in the published protocol) results in removing noise and closing gaps in the microglial processes. Finally, we skeletonize the image using the Skeletonize and Analyze Skeleton functions in ImageJ.

### Primary neuron culture and treatments

Primary neuron cultures were prepared following an established protocol [32]. Briefly, early postnatal (P0-P1) mouse (C57BL/6j) hippocampi and cortices were isolated, rinsed, trypsinized, dissociated and plated on prepared poly-L-lysine coated coverslips. Within two days after plating, cytosine arabinoside (araC; 1-b-d-arabinofuranosylcytosin) is added to inhibit proliferating non-neuronal cells resulting in a predominantly neuronal cell population. Cultures were grown for 16 days in vitro (DIV) at 37°C in humidified 5% CO_2_ incubator prior to any treatment. DIV 16 cultures were treated with 25 ng/mL recombinant mouse IL-6 (Biolegend) and collected in LDS/RA solution 12 h after treatment. Collected cell lysates in LDS/RA were sonicated and boiled before use in western immunoblot assay.

### Adult neuron isolation and treatments

Dissociation of adult mouse brains was performed using the “Adult Brain Dissociation Kit for mouse and rat” (Miltenyi Biotec, 130-107-677) and “Neuron Isolation Kit for mouse” (Miltenyi Biotec, 130-115-389) following the user manual, in combination with the gentleMACS Dissociator (Miltenyi Biotec). Mice were anesthetized and the brains removed then cut into 8 sagittal slices. Brain slices were homogenized using the gentle MACS Dissociator in C Tubes (Miltenyi Biotec, 130-093-237) with the recommended volume of enzyme mixes. Tissue was processed with no modification to the protocol in the kit. Isolated neurons from the adult mouse brains were cultured for one week changing half the media (MACS Neuro Medium containing 2% MACS Neuro Brew-21,1% penicillin/streptomycin, and 0.5 mM l-glutamine) every other day. One week after neuronal isolation, neurons were treated with 25 ng/mL recombinant mouse IL-6 (Biolegend) and collected in LDS/RA solution 12 h after treatment. Collected cell lysates in LDS/RA were sonicated and boiled before use in western immunoblot assay.

### Statistics

Unless otherwise indicated, comparisons between the two groups were done via unpaired *t* test. Comparisons between multiple treatment groups were done via one-way or two-way analysis of variance (ANOVA) with Sidak’s multiple comparison post hoc test. All statistical analyses were performed using GraphPad Prism® (Version 8).

## Results

### Genetic deletion of *Il10* promotes tau hyperphosphorylation after peripheral LPS administration

**Fig. 1:**
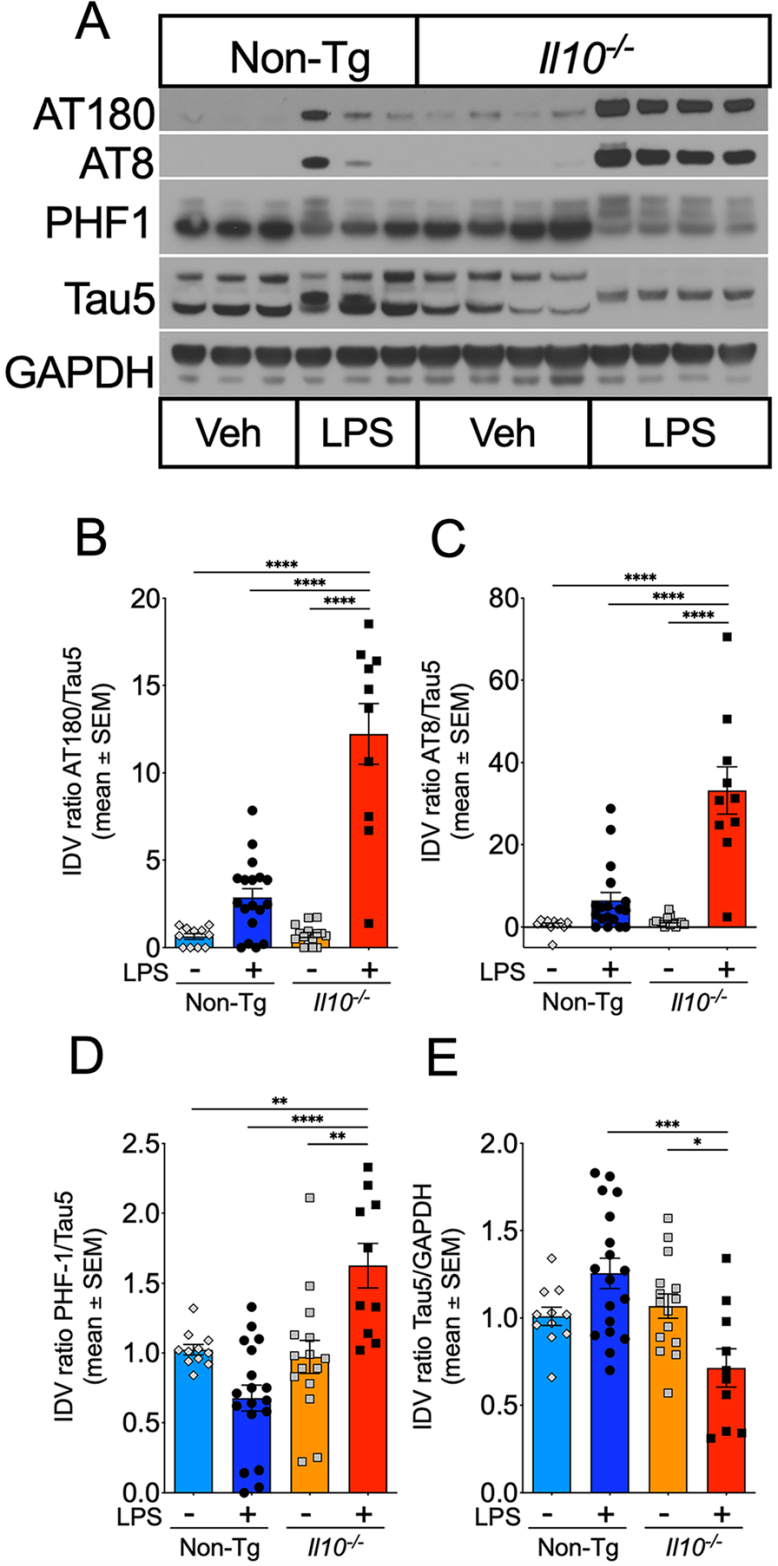
Genetic deletion of *Il10* promotes tau hyperphosphorylation after LPS injection. Non-Tg or *Il10*^*−*/*−*^ mice injected with LPS or Veh were sacrificed 24 h after LPS injection and brain tissue was extracted for analysis. **A** Western immunoblot (WB) of hippocampal (Hp) homogenates to assess levels of phosphorylated AT8, AT180, and PHF-1 tau epitopes. Glyceraldehyde 3-phosphate dehydrogenase (GAPDH) was analyzed as a protein loading control. **B**–**D** Quantification of immunoreactive bands as a ratio of pTau (AT180, AT8, and PHF-1) over total tau (Tau5). **E** Quantification of immunoreactive bands as a ratio of Tau5 over GAPDH. Data shown are mean + SEM of integrated density value (IDV) ratio; * *p* < 0.05, ** *p* < 0.01, *** *p* < 0.001; two-way ANOVA with Sidak’s multiple comparison test; *n* = 10–18 mice; each individual data point shows an individual mouse sample

Abnormal hyperphosphorylation of tau is regarded as an early hallmark of AD [33, 34]. Well-characterizedAD-relevant phosphorylated tau epitopes at Ser202/Thr205, Thr231, and Ser396/Ser404 detected by AT8, AT180, and PHF-1 antibodies, respectively, can serve as markers for tau pathology [35–37]. Protein analysis via Western blot of hippocampal lysates from *Il10*^*−*/*−*^ and Non-Tg mice was performed to determine the levels of tau phosphorylation at AT8, AT180, and PHF-1 epitopes 24 h following one dose of intraperitoneal LPS injection (Fig. 1A–D). No significant differences were found in the basal levels of phosphorylated tau (pTau) or total tau between *Il10*^*−*/*−*^ and Non-Tg mice treated with Veh. However, 24 h following LPS-injection, both the *Il10*^*−*/*−*^ and Non-TgLPS-treated groups had increased AT8+ and AT180+ pTau. Importantly, the *Il10*^*−*/*−*^ LPS mice had significant increases in AT8+, AT180+, and PHF-1+ pTau compared to the Non-Tg LPS mice (Fig. 1B–D). Notably, total tau levels were significantly reduced in *Il10*^*−*/*−*^ mice after LPS administration (Fig. 1E) compared to *Il10*^*−*/*−*^ Veh mice and Non-TgLPS-treated mice. These data suggest that deficiency of IL-10 exacerbates hyperphosphorylation of tau in the hippocampus within 24 h of LPS administration.

**Fig. 2:**
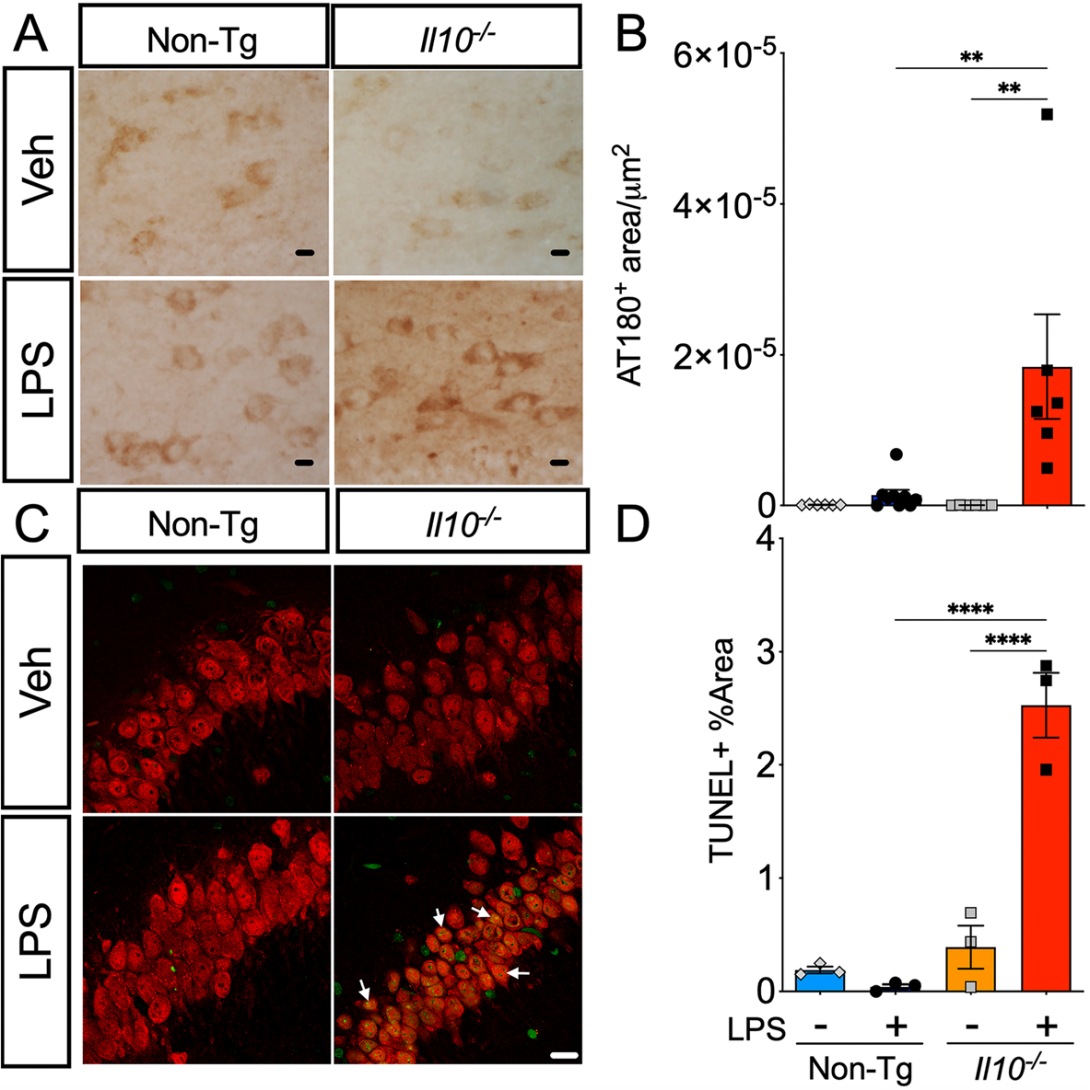
LPS-treated *Il10*^*−*/*−*^ mice have increased neuronal p-tau and increased neuronal apoptotic cell death. Brain sections from Non-Tg or *Il10*^*−*/*−*^ mice described above. **A**, **B** Immunohistochemistry (IHC) for endogenous pTau probed with AT180 antibody; 40× magnification; scale bar = 10 μm. **A** Representative sections from the dentate gyrus (DG) region of interest (ROI). **B** Quantification of AT180+ area. Data shown are mean ± SEM of area/μm^2^ from the standardized averages of three repeat trials; * *p* < 0.05, ** *p* < 0.01, *** *p* < 0.001; two-way ANOVA with Sidak’s multiple comparison test; *n* = 4–9 mice/group. **C**, **D** TUNEL immunofluorescence assay co-stained with NeuN antibody (arrows) to identify neurons in the Hp CA3 region; 63× magnification; scale bar = 10 μm. **D** Quantification of TUNEL+ area (green). Data shown are mean ± SEM of % area TUNEL+ cells in CA3 ROI; * *p* < 0.05, ** *p* < 0.01; *** *p* < 0.001; 2-way ANOVA; *n* = 3 mice/group; each individual data point shows an individual mouse sample

To verify the spatial distribution of phosphorylated tau in different sub-regions within the dentate gyrus of the mouse brains, immunohistochemistry (IHC) of sagittal brain sections was performed. Quantification of AT180+ area, 24 h post-LPS treatment, confirmed significantly enhanced pTau in the dentate gyrus of *Il10*^*−*/*−*^ mice compared to Non-Tg mice (Fig. 2A, B). The morphology of the AT180+ area resembled neuronal cell bodies rather than astrocytes or oligodendrocytes, which can also abnormally harbor pTau [38–40]. These results confirm significantly increased phosphorylated tau epitopes in hippocampal neurons of *Il10*^*−*/*−*^ mice.

### LPS-treated *Il10*^*−*/*−*^ mice show signs of neurodegeneration

We speculated that a loss of total tau mentioned above is most likely due to neuronal death since tau is highly enriched in neurons [4]. To evaluate the possibility of neuronal cell death within the hippocampi of the LPS-injected mice, neuronal cell death was examined using TUNEL assay. There was notable detection of TUNEL positive cells (green) colocalized with NeuN+ neurons (red) that appeared to have yellowish nuclei (arrows) in the CA3 hippocampal regions of *Il10*^*−*/*−*^ LPS mice compared to the Veh and Non-Tg LPS groups (Fig. 2C). Quantification confirmed the increased number of TUNEL+ cells in the neuronal layer of *Il10*^*−*/*−*^ LPS mice compared to the Veh and Non-Tg LPS groups (Fig. 2D). These data corroborate neuronal cell death in *Il10*^*−*/*−*^ LPS mice, which is consistent with the significant reduction in neuron-enriched tau protein.

### *Il10*deficiency enhances LPS-induced p38 MAPK activation

**Fig. 3:**
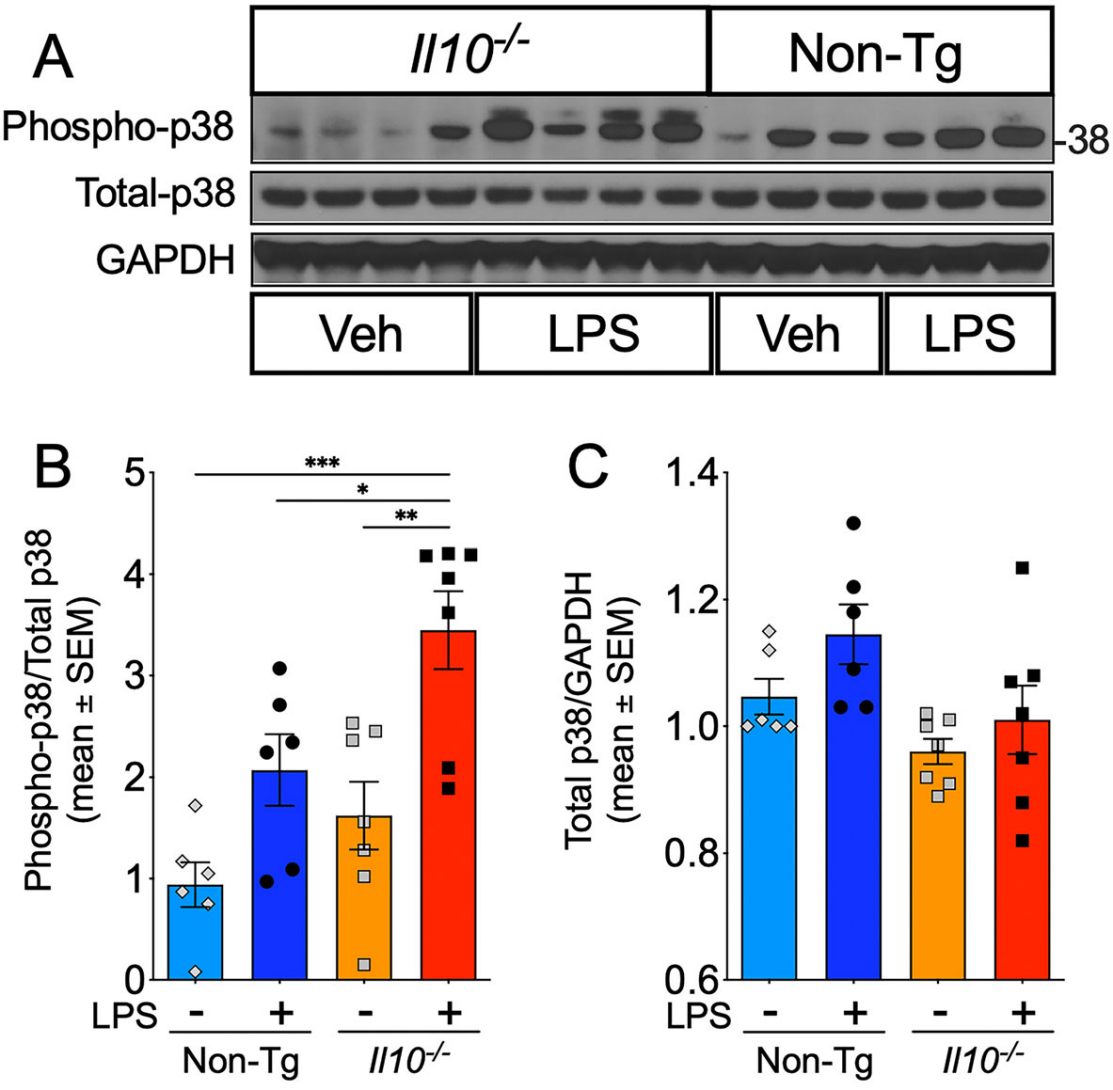
*Il10* deficiency significantly enhances p38 MAPK activation after LPS injection. WB of Hp homogenates from Non-Tg or *Il10*^*−*/*−*^ mice described above. **A** Levels of (activated) phospho-p38 MAPK relative to (total) p38 MAPK; GAPDH protein loading control. Each lane represents an individual mouse sample. **B**, **C** Quantification of immunoreactive bands as ratio of phospho-p38 over total-p38 (**B**) and total-p38 relative to GAPDH (**B**). Data shown are mean ± SEM of IDV ratio; * *p* < 0.05; ** *p* < 0.01; *** *p* < 0.001; two-way ANOVA with Sidak’s multiple comparison test; *n* = 6–7 mice/group

Activated p38 MAPK is a member of the MAPK family and key signal transduction protein involved in CNS inflammation [41] and implicated in tau phosphorylation [12, 42–46]. To determine if activated p38 MAPK was elevated in the *Il10*^*−*/*−*^ mice, we examined levels of phosphorylated p38 MAPK (phospho-Thr180/Tyr182) in the hippocampal lysates (Fig. 3). Basal levels of phospho-p38 were no different in *Il10*^*−*/*−*^ mice vs Non-Tg mice and LPS administration did not significantly elevate levels of active p38 MAPK in LPS-injectedNon-Tg mice (Fig. 3A, B). However, robust and statistically significant phospho-p38 MAPK activation was observed in LPS-injected*Il10*^*−*/*−*^ mice compared to the LPS-injectedNon-Tg control group (Fig. 3A, B). No statistically significant differences in the total p38 ratio over GAPDH were observed in either genotypes or treatment groups when assessed by two-way ANOVA (Fig. 3C). These data suggest IL-10 deficiency alters active forms of p38 MAPK and, in the context of an inflammatory response, correlates with enhanced tau phosphorylation and neurodegeneration.

### *Il10*-deficient microglia display activated morphology with LPS treatment

**Fig. 4:**
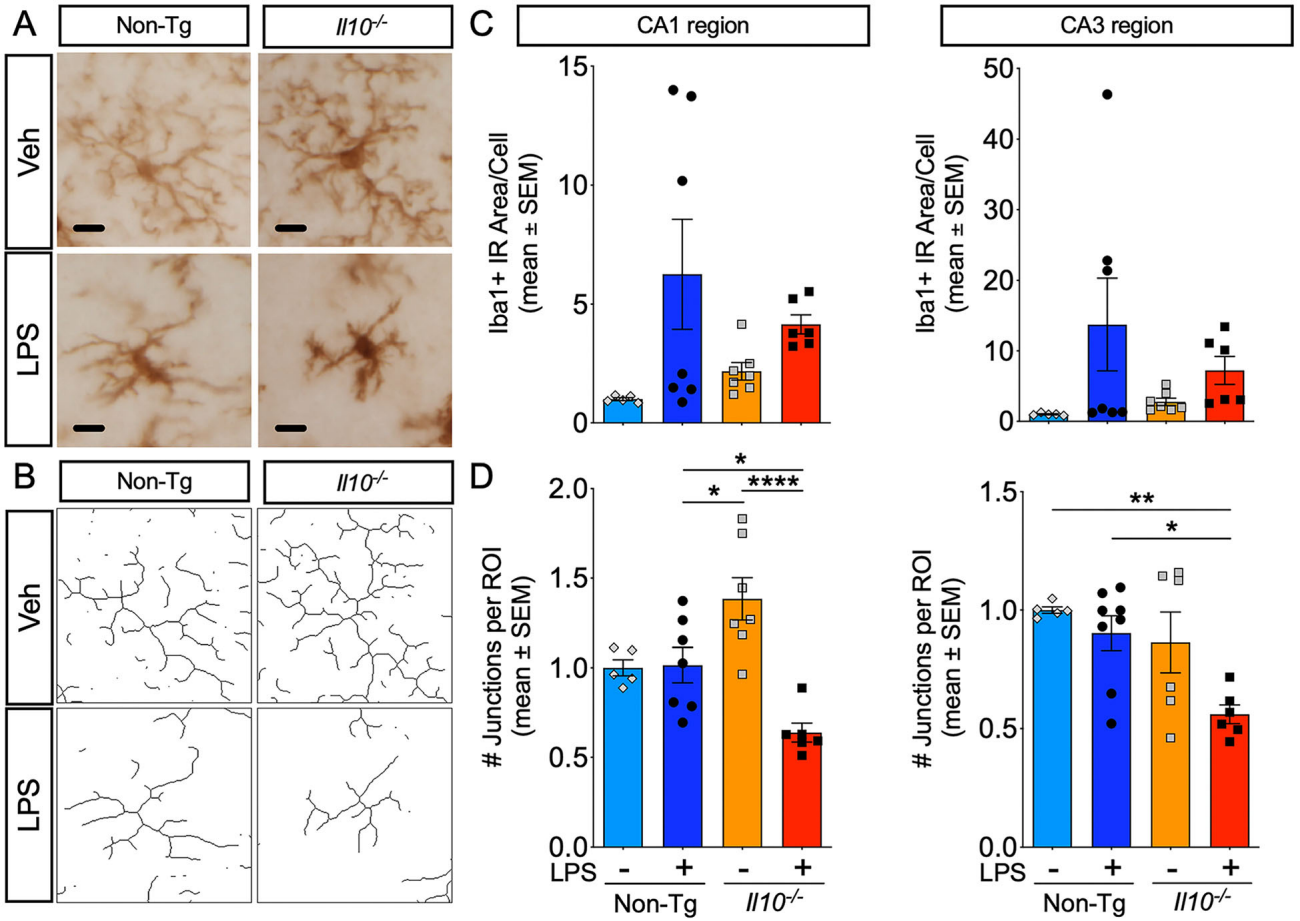
*Il10* deficiency promotes a proinflammatory microglia morphology after LPS injection. IHC to visualize microglial morphology in CA1 and CA3 regions from Non-Tg or *Il10*^*−*/*−*^ mice described above; 40× images were quantified; scale bar = 10 μm. **A** Representative Iba1+ microglia from CA1 ROI. **B** Representative skeletonized microglia (derived from **A**). **C**, **D** Quantification of Iba1+ immunoreactive (IR) area (**C**) and the number of junctions per ROI (**D**). Data shown are mean ± SEM of normalized values; * *p* < 0.05, ** *p* < 0.01; *** *p* < 0.001; two-way ANOVA with Sidak’s multiple comparison test; *n* = 5–7 mice/group. Each individual data point represents an individual mouse sample

Next, we wanted to assess the microglial phenotype in *Il10*^*−*/*−*^ mice after LPS administration to reassert the role of reactive microglia in driving pTau. Brain sections were stained with Iba1 to visualize microglial morphology (Fig. 4A) and a skeleton analysis was performed to quantify the branching (data not shown) and junctions of microglial processes in the hippocampus (Fig. 4B). We observed that *Il10*^*−*/*−*^ Veh mouse brains already had a slightly elevated, though not statistically significant, Iba1+ area compared to Non-Tg Veh controls. Interestingly, the *Il10*^*−*/*−*^ Veh microglia in the CA1 region had slightly increased branching (data not shown) and significantly increased the number of junctions compared to the other groups (Fig. 4D). After LPS administration, we observed that the Iba1+ area increased in both groups, suggesting a more activated phenotype than vehicle-treated respective control (Fig. 4C). Notably, there was no significant enhancement of Iba1 expression attributed to IL-10 deficiency after LPS administration. However, analysis of the skeletonized branching of the microglia revealed that *Il10*^*−*/*−*^ microglia had significantly reduced branching (data not shown) and decreased the number of junctions compared to Non-Tg microglia post-LPS (Fig. 4D). This suggests a more amoeboid, highly activated phenotype resembling pro-inflammatory microglia in the LPS-treated*Il10*^*−*/*−*^ mice brains compared to LPS-treatedNon-Tg controls.

### *Il10* deficiency significantly enhanced inflammatory cytokines

**Fig. 5:**
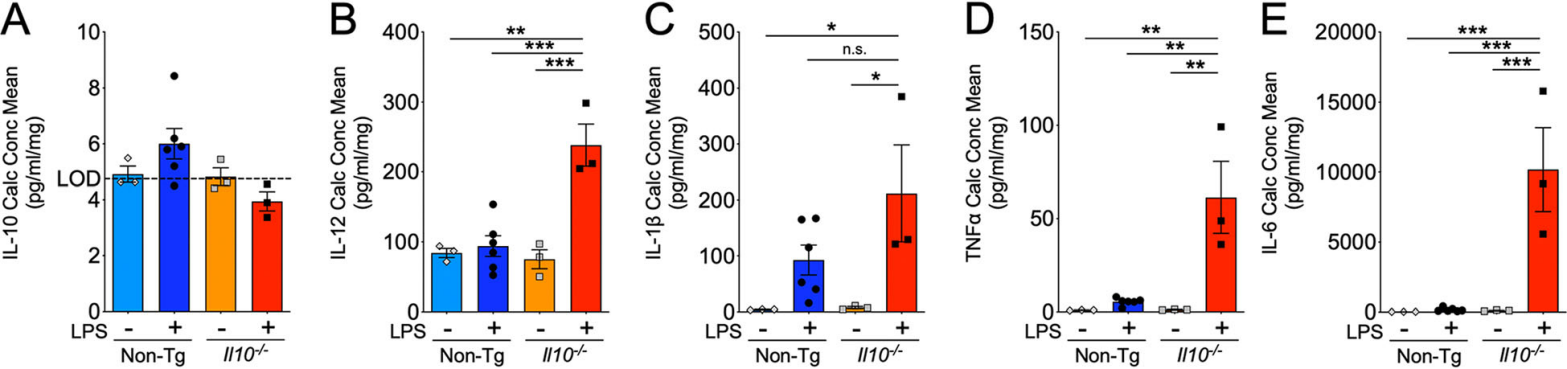
*Il10* deficiency significantly increased inflammatory cytokine response. Multiplex electrochemiluminescent immunoassay of brain tissue homogenates from Non-Tg or *Il10*^*−*/*−*^ mice described above. **A**–**E** Quantification of IL-10 (**A**), IL-12 (**B**), IL-1β (**C**), TNFα (**D**), and IL-6 (**E**) cytokines in Veh and LPS-treated mouse brains. Data shown are mean ± SEM of calculated concentration mean (pg/mL/mg); * *p* < 0.05, ** *p* < 0.01; *** *p* < 0.001; two-way ANOVA with Sidak’s multiple comparison test; *n* = 3–6 mice/group. Hash lines indicate the level of detection (LOD) provided in reagent documentation

Cytokine levels in cortical tissue lysates from *Il10*^*−*/*−*^ and Non-Tg mouse brains were examined to confirm the pro-inflammatory phenotype. First, basal levels of IL-10 were below the level of detection in both groups prior to LPS. Basal levels of IL-12, IL-1β, and TNFα were low and not significantly different in *Il10*^*−*/*−*^ mice vs Non-Tg controls. Slightly elevated levels of IL-10 were still detectable in a number of LPS-treatedNon-Tg mice 24 h after the LPS challenge whereas IL-10 remained below background levels of detection in LPS-treated*Il10*^*−*/*−*^ mice (Fig. 5A). Additionally, the Non-Tg group had elevated levels of IL-1β, TNFα, and IL-6 after LPS administration (Fig. 5C–E). In comparison, *Il10*^*−*/*−*^ mice had significantly enhanced levels of IL-12, TNFα, and IL-6 relative to Non-Tg controls (Fig. 5B, D, E). Out of all four pro-inflammatory cytokines studied, a thousand-fold elevated increase in IL-6 levels that remained elevated at 24 h post-LPS injection in LPS-treated*Il10*^*−*/*−*^ mice was very striking (Fig. 5E). Surprisingly, IL-1β was not found to be significantly elevated in the LPS *Il10*^*−*/*−*^ mice compared to LPS-treatedNon-Tg controls at this 24 h timepoint (Fig. 5B).

### Direct stimulation of primary neurons with IL-6 induces tau hyperphosphorylation

**Fig. 6:**
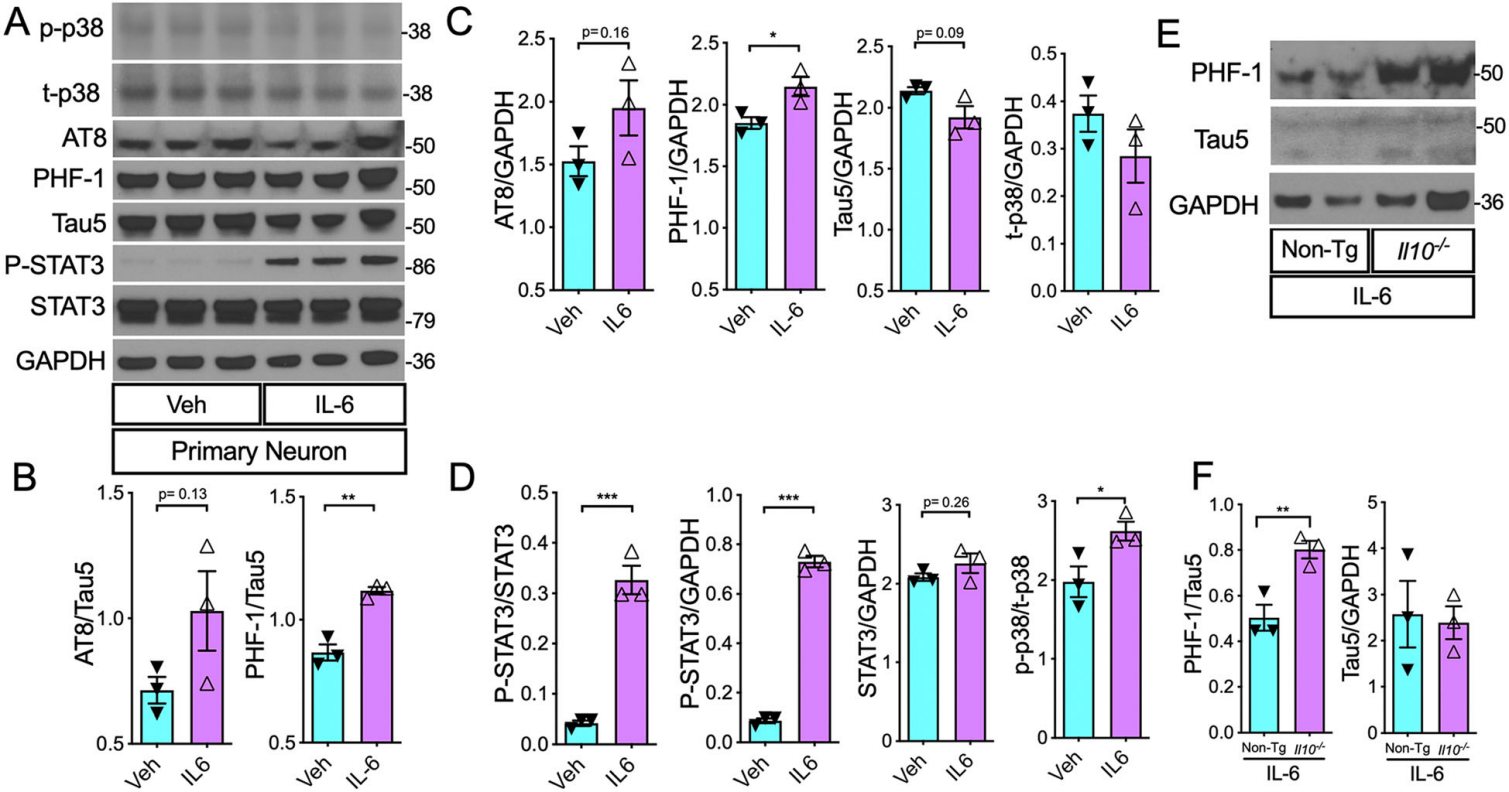
IL-6 stimulation increased p-tau in primary mouse neurons, in vitro. **A** WB of cell lysates from primary neuronal cell cultures directly treated with recombinant IL-6 protein (25 ng/mL) or Veh. **B** Quantification of pTau over total tau, AT8/Tau5, and PHF-1/Tau5 ratios. **C** Quantification of pTau (AT8/GAPDH and PHF-1/GAPDH) and total tau (Tau5/GAPDH) relative to loading control. **D** Quantification of (activated) phospho-STAT3 (P-STAT3) relative to (total) STAT3. Quantification of P-STAT3 and STAT3 relative to GAPDH. **E**, **F** WB of cell lysates of adult neurons from non-transgenic (Non-Tg) and IL-10-deficient mice (*Il10*^*−*/*−*^) show significantly higher PHF-1/Tau5 ratio in IL-10-deficient neurons treated with 25 ng/mL recombinant IL-6 compared to Non-Tg neurons treated with the same dose of IL-6. Data shown are mean ± SEM of IDV ratio; * *p* < 0.05, **p < 0.01; *** *p* < 0.001; unpaired *t* tests; *n* = 3 neuronal culture wells/treatment group

Since IL-6 was expressed by more than a thousand-fold increase in LPS-treated*Il10*^*−*/*−*^ mice compared to LPS-treatedNon-Tg controls, we evaluated the potential role of IL-6 on tau phosphorylation in murine primary neurons. First, we treated mouse primary neuronal cultures derived from P0 C67BL/6j pups with recombinant IL-6 and performed Western blot analysis for phospho-tau in the cell lysates. We verified that activated STAT3 (phospho-tyr705) levels were significantly elevated in IL-6-treated neurons (Fig. 6A–D) indicating expected intracellular response downstream of IL-6 signaling. Next, we observed a modest increase in the AT8/Tau5 and AT8/GAPDH ratios in IL-6-treated neurons compared to vehicle-treated controls. Importantly, PHF-1/Tau5 and PHF-1/GAPDH ratios were significantly higher in IL-6 stimulated neurons relative to Veh controls (Fig. 6A–D). Interestingly, similar to the trends we observed with the in vivo results, there was a modest reduction in total tau (Tau5/GAPDH ratio) and an increase in p-p38/total-p38 MAPK ratio in the IL6-treated neurons (Fig. 6A–D). Furthermore, stimulation of neurons derived from adult IL-10 knockout mice with IL-6 showed significantly elevated levels of PHF-1/Tau5 ratio compared to adult non-transgenic neurons treated with IL-6 (Fig. 6E–F). This suggests that IL-6 is sufficient to promote increased tau phosphorylation within neurons and it is enhanced in neurons deficient in IL-10.

## Discussion

In this study, we demonstrate that deficiency of IL-10 was sufficient to increase tau hyperphosphorylation after only 24 h of a moderate dose of LPS treatment. We observed tau phosphorylation was enhanced in *Il10*^*−*/*−*^ mice treated with LPS. We detected reduced total tau and increased neuronal cell death in hippocampi of LPS-treated*Il10*^*−*/*−*^ mice. We confirmed that an important tau kinase, p38 MAPK, was activated in hippocampal tissue of *Il10*^*−*/*−*^ mice after LPS administration and significantly enhanced in the LPS-treated*Il10*^*−*/*−*^ mice compared to LPS-treatedNon-Tg controls. *Il10*^*−*/*−*^ microglia had morphological features of activated amoeboid cells, along with upregulation of pro-inflammatory cytokines, IL-12, TNFα, and IL-6, after LPS treatment. Particularly, IL-6 was substantially increased in the LPS-treated*Il10*^*−*/*−*^ mice and we demonstrated that direct stimulation with IL-6 is sufficient to induce tau phosphorylation in a primary mouse neuronal culture. These data substantiate a role for IL-10 in regulating the inflammatory response to an immune challenge through TLR4 signaling that involves p38 MAPK, microglial activation, and excessive pro-inflammatory cytokine expression, which results in hyperphosphorylation of tau.

IL-10 dysregulation is highly relevant to AD in a number of ways. First, there are single nucleotide polymorphisms (SNPs) in the *Il10* promoter associated with increased risk of AD [22, 47]. For example, the ATA haplotype (−1082A/−819 T/−592A) of the IL-10 gene promoter region is associated with susceptibility to AD [20, 48]. Particularly, an “A” allele at position 1082 has been associated with low production of IL-10 [49] and is implicated in promoting mild cognitive impairment (MCI) to AD [19, 47]. In line with the association of low IL-10 production with AD, patients with faster rates of decline in their mini-mental state exams (MMSE) were shown to have significantly lower production of IL-10 upon ex vivo peripheral blood mononuclear cell (PBMC) stimulation, suggesting that IL-10 expression could be protective in the slow declining population [50]. Our study corroborates the concept of IL-10 dysfunction leading to an increased pathology related to AD.

Second, IL-10 regulates microglial phenotype and immune processes, which are implicated in AD. According to a recent genome-wide association study (GWAS) by the International Genomics of Alzheimer’s Project, some of the most influential risk factors for AD compared to controls were in genotypes associated with microglia and the immune system [51, 52]. Furthermore, animal studies have shown that reactive microglia are sufficient to drive tau pathology [12, 53] but the role of pro- and anti-inflammatory mediators on microglial phenotype and function is still being understood. Dysregulated IL-10 expression has been shown to alter microglial phenotype through both overexpression and deficiency [54, 55] but there are still conflicting data on how IL-10 influences microglial function. For example, in the context of amyloid plaque clearance, it has been shown that IL-10 dampens β-amyloid clearance and IL-10 deficiency permits β-amyloid phagocytosis by microglia [24, 56]. On the other hand, a recent in vitro study suggests that IL-10 promotes phagocytosis dependent on the triggering receptor expressed on myeloid cells 2 (TREM2) in microglia [57]. IL-10 deficiency, in our LPS model, promoted a highly activated, pro-inflammatory microglial phenotype. Interestingly, the microglia in our Veh-treated*Il10*^*−*/*−*^ mice, which are deficient in IL-10 throughout their life, seemingly had an intermediate stage of activation (increased Iba1 and hypertrophic processes) in the hippocampus CA1, but not the CA3, region. Thus, discerning the effects of dysregulated IL-10 on AD may require an understanding of intermediate activation states of microglia, the microglial microenvironment, and the dosage as well as the timing of IL-10.

Finally, dysregulation of cytokines, including IL-10, is implicated in AD. For example, inflammasome signaling molecules are increased in AD brains [58] and colocalize with microglia around plaques [8]. Furthermore, patients with advanced rates of progression from MCI to dementia in AD have elevated levels of pro-inflammatory cytokines in the CSF [59]. Although IL-10 is also found to be upregulated in the CSF of AD patients [60], it is concomitant with elevation in pro-inflammatory cytokine levels. This is in line with the role of IL-10 in negative feedback inhibition in response to inflammatory stimuli [17, 61]. On the same note, we saw that the absence of IL-10 lead several cytokines, including TNFα and IL-6, to run rampant after the LPS challenge. IL-10 signaling through the IL-10 receptor in microglia involves activation of the JAK/STAT/SOCS pathway [62] resulting in IL-10 induced genes. This includes dual-specificity phosphatases (DUSP), which downregulates p38 MAPK activation in myeloid cells [63, 64]. In line with this, we saw that the absence of IL-10 lead to increased p38 MAPK activation. IL-10 has been shown to regulate the expression of a number of pro-inflammatory cytokines in microglial cultures and brain tissue [15, 65, 66]. This includes IL-1β, which can peak at 5 h in the brain after peripheral LPS administration in rodents [66] but has complex endotoxin-dependentIL-1β maturation kinetics depending on extracellular ATP release [67]. Interestingly, we observed that IL-1β cytokine expression did not remain significantly elevated in *Il10*^*−*/*−*^ mice compared to Non-Tg mice at 24 h after LPS administration. This may suggest another compensatory mechanism limited the ongoing expression of IL-1β within 24 h in our model. However, we do not know the relative expression levels of IL-1β at earlier timepoints and cannot rule out a role for IL-1β in promoting tau hyperphosphorylation in this model. Potentially, there are multiple routes leading to AD-relevant tau phosphorylation if left unregulated. In our study, TNFα and IL-6, were particularly elevated by LPS in the absence of IL-10. Interestingly, IL-6 remained significantly elevated in the *Il10*^*−*/*−*^ mice, which alludes to clinical correlations between genotypes associated with low IL-10 and high IL-6 production as a risk factor for AD [21, 68, 69]. Therefore, we explored the potential for IL-6 to promote tau phosphorylation in neurons by directly stimulating neuron-enriched primary cultures with recombinant IL-6.IL-6 stimulation was sufficient to induce tau phosphorylation in primary hippocampal neurons consistent with a previous study that showed IL-6 stimulation increased neuronal pTau [70]. Interestingly, the study by Quintanilla et al., showed that treatment of primary hippocampal rat neurons with 5 ng/mL IL-6 significantly increased phosphorylated tau (AT8/Tau5 ratio) and lowered unphosphorylated tau (Tau1/Tau5 ratio) after a 48-h incubation. In our study, treatment of primary hippocampal mouse neurons with 25 ng/mL of IL-6 for 12 h significantly increased tau phosphorylation on the Ser396/Ser404 (PHF-1) epitope and total tau (Tau5/GAPDH) was significantly decreased. These data support a role for dysregulated cytokines, including IL-10 and IL-6, in promoting AD-relevant tau phosphorylation. It is important to note that our results do not show elevated cytokine levels in IL-10 knockout mice with LPS is cell-autonomous to microglia. As such, at this stage, it is still correlational and require single cell RNA sequencing and profiling of microglia based on their cytokine expression phenotype. Performing such studies is beyond the scope of the present study and our future studies will determine if disease-associated microglia (DAMs), interferon-responsive microglia (IRMs), and other types of microglia relevant to AD/tauopathies are specifically altered in IL-10 knockout mice brains with LPS challenge.

**Fig. 7:**
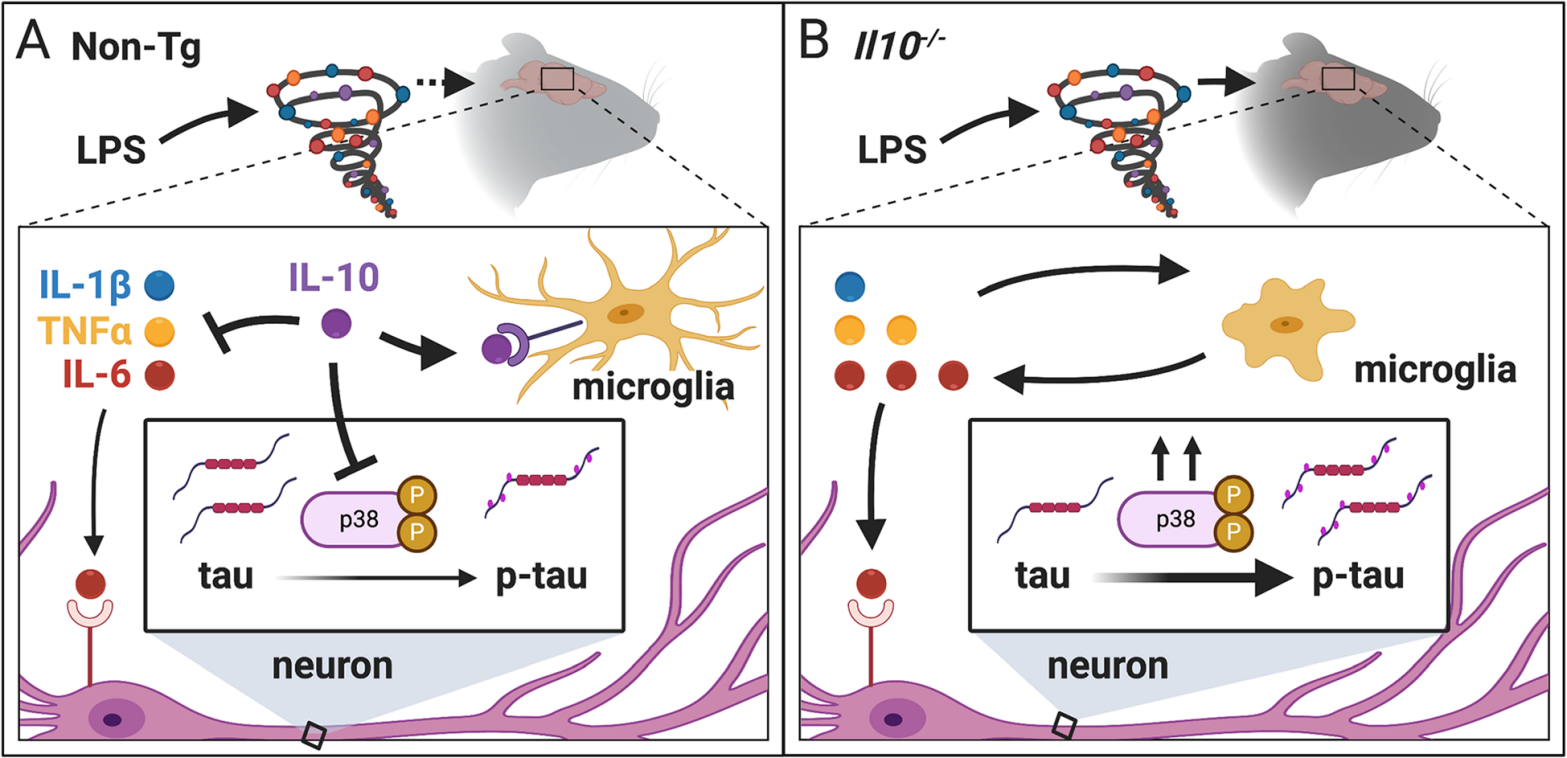
Proposed model for IL-10 regulation of neuroinflammation and tau pathology. **A** In non-transgenic (Non-Tg) mice, we observed an increase in proinflammatory cytokines, including IL-6, TNFα, and IL-1β, with a concomitant increase in anti-inflammatory cytokine, IL-10. IL-10 provides negative feedback to dampen cytokine production, microglial activation, and p38 MAPK activation resulting in only low levels of tau phosphorylation. **B** In IL-10-deficient mice (*Il10*^*−*/*−*^), the absence of IL-10 leads to enhanced cytokine production, especially for IL-6 and TNF, microglia are more active and more amoeboid, and p38 MAPK levels are also enhanced. Tau phosphorylation is exacerbated in neuronal bodies of the *Il10*^*−*/*−*^ brains. Created with BioRender.com

The role that inflammation plays and whether it contributes to AD has increasingly become an important area of research due to increased evidence that microglial activation and abnormal expression of cytokines are associated with AD. Understanding inflammatory pathways that promote tauopathy is necessary to find novel targets for AD. Here, we provide evidence that IL-10 deficiency enhances a pro-inflammatory environment in the brain after a peripheral immune challenge leading to hyperphosphorylation of tau on AD-relevant epitopes. Our model involves inducing inflammation through TLR4 signaling using LPS as the stimulus. Immune activation through TLR4 is relevant in AD as amyloid beta (Aβ), is known to trigger TLR4 signaling to secrete inflammatory factors [71, 72]. TLR4 can also be triggered by danger-associated molecular patterns (DAMPs) due to neuronal injury and other tissue damage [73]. Therefore, this model pertains to TLR4 signaling promoting tauopathy and is not strictly pertinent to immune response due to infection. Moreover, several recent reports implicate the endotoxin hypothesis driving neurodegeneration [74–76]. Whether the moderate dose of peripheral LPS used in our study leads to direct TLR4 stimulation within the brain parenchyma or induces a cytokine storm to trigger brain inflammation, the result in our study is that microglia become highly activated and the brain is flooded with pro-inflammatory molecules that persist after 24 h in IL-10-deficient mice. Our data is consistent with a model (Fig. 7), whereby IL-10 has an important role in limiting an ongoing inflammatory response leading to tau hyperphosphorylation relevant to AD.

## Conclusions

Our study shows that IL-10 deficiency is sufficient to promote tau hyperphosphorylation and dysregulated neuroinflammation in the context of an acute peripheral inflammatory insult. Genetic deletion of *Il10* promoted enhanced pro-inflammatory cytokine expression, activated microglial morphology, and evidence of neuronal loss. Exacerbated neuroinflammation in the IL-10-deficient environment corresponded with increased tau-kinase p38 MAPK activation and significantly increased tau phosphorylation. These data support the significance of IL-10 in modulating pro-inflammatory microglial activation, dysregulated cytokine production, and hyperphosphorylation of AD-relevant tau epitopes.

## Data Availability

The datasets used and/or analyzed during the current study are available from the corresponding author on reasonable request.

## Abbreviations

AD: Alzheimer’s disease
CNS: Central nervous system
DG: Dentate gyrus
GAPDH: Glyceraldehyde 3-phosphate dehydrogenase
Hp: Hippocampus
Iba-1: Ionized calcium-binding adapter molecule 1
IDV: Integrated density value
IHC: Immunohistochemistry
IL-1β: Interleukin-1β
IL-10: Interleukin-10
*Il10*^*−*/*−*^: *Il10-*deficient mice
IL-12: Interleukin-12
IL-6: Interleukin-6
IR: Immunoreactive
LOD: Level of detection
LPS: Lipopolysaccharide
MAPT: Microtubule-associated protein tau
MCI: Mild cognitive impairment
MMSE: Mini-mental state exams
MSD: Meso Scale Discovery multiplex
NeuN: Neuronal nuclei
Non-Tg: Non-transgenic
PBMC: Peripheral blood mononuclear cell
p38-MAPK: p38 mitogen-activated protein kinase
p38α-MAPK: p38 mitogen-activated protein kinase type α
p-Tau: Hyper-phosphorylated tau
SNP: Single nucleotide polymorphism
SOCS: Suppressor of cytokine signaling
TLR4: Toll-like receptor 4
TREM2: Triggering receptor expressed on myeloid cells 2
TNF-α: Tumor necrosis factor α
TUNEL: Terminal deoxynucleotidyl transferase dUTP nick end labeling
